# 4-*n*-Butylresorcinol-Based Linear and Graft Polymethacrylates for Arbutin and Vitamins Delivery by Micellar Systems

**DOI:** 10.3390/polym12020330

**Published:** 2020-02-05

**Authors:** Justyna Odrobińska, Łukasz Mielańczyk, Dorota Neugebauer

**Affiliations:** 1 Department of Physical Chemistry and Technology of Polymers, Faculty of Chemistry, Silesian University of Technology, 44-100 Gliwice, Poland; justyna.odrobinska@polsl.pl; 2 Department of Histology and Cell Pathology, Faculty of Medical Sciences in Zabrze, Medical University of Silesia, 40-055 Katowice, Poland; lmielanczyk@sum.edu.pl

**Keywords:** 4-*n*-butylresorcinol, graft copolymers, “click” chemistry, micellar systems, amphiphilic properties, vitamin delivery, cosmetology

## Abstract

A novel initiator, bromoester modified 4-*n*-butylresorcinol (4nBREBr_2_), was prepared and utilized in controlled atom transfer radical polymerization (ATRP) to obtain three series of amphiphilic copolymers. The V-shaped copolymers of methyl methacrylate (MMA), 2-hydroxyethyl methacrylate (HEMA), and poly(ethylene glycol) methyl ether methacrylate (MPEGMA), abbreviated to P(HEMA–*co*–MMA), P(HEMA–*co*–MPEGMA), and P(MMA–*co*–MPEGMA), were synthesized. Moreover, P((HEMA–*graft*–PEG)–*co*–MMA) graft copolymers were prepared by combining the pre-polymerization modification of HEMA and a “click” reaction using a “grafting onto” approach. All copolymers could form micelles with encapsulated active substances (vitamin C (VitC), vitamin E (VitE), arbutin (ARB)), which are used in cosmetology. In vitro studies carried out in a PBS solution (pH 7.4) demonstrates that in most cases the maximum release of active substance was after 1 h. The polymeric systems presenting satisfactory encapsulation characteristics and release profiles are attractive micellar carriers of cosmetic substances, which show a positive effect on the skin condition.

## 1. Introduction

4-*n*-Butylresorcinol (4nBRE) is a derivative of retinol (RET, commonly known as vitamin A), which is widely used in cosmetic products such as anti-wrinkle creams and used to reduce discoloration. 4nBRE has already been applied in the synthesis of hydroxyacetophenone derivatives [1], derivatives with benzenepropanoic acid [2], new benzopyrones with bacteriostatic activity [3], naturally occurring 6-acyl-7-methoxycoumarins [4], and pyrazole analogues of isoflavones [5]. Applications of 4nBRE in cosmetology show it to be an effective inhibitor of tyrosinase activity and melanin production for the treatment of hyperpigmentation [6,7,8,9,10]. Additionally, published laboratory studies show much higher effectiveness of 4nBRE when compared to arbutin (ARB) and hydroquinone [9] regarding depigmentation effects. Its efficacy and rapid action have also been confirmed in clinical tests, where encapsulated liposomes of 4nBRE were used as a 0.1% component of a cream [6].

In delivery systems, a critical aspect is the selection of a suitable carrier [11]. Recently, polymeric micelles gained great attention as carriers. The literature broadly reports the use of micelles as effective delivery systems for anti-cancer drugs (doxorubicin [12,13], paclitaxel [14,15]), anti-inflammatory (e.g., indomethacin [16,17,18]), or antibacterial drugs (erythromycin [19]) as well as for the active substances used in cosmetology (vitamin K [20,21], vitamin D [22], vitamin A [23,24,25,26], and vitamin E [27,28]). Current trends in polymeric micelles are focused on developing multifunctional [29,30], stimuli-sensitive for targeted drug delivery [31,32,33] and replacement of synthetic compounds by natural ones [34,35]. In many cases, the azide-alkyne cycloaddition “click” reaction has been used to synthesize the micelle-forming polymer-carriers, such as thermosensitive polypeptides, by grafting of azide functionalized oligoethylene glycol onto poly(γ-propargyl-l-glutamate) [36], thermosensitive star-comb and miktoarm polymethacrylates [37], light-sensitive polycarbonates containing spiropyran units [38], or poly(ethylene glycol–*co*–lactic acid–*co*–carbonate) with a terminal biotin unit conjugated by pH-sensitive oxime linker [39].

Here we focus on biologically active substances, especially vitamin initiators, which after incorporation into the polymers increase their biocompatibility and transport effectiveness of their encapsulated components. Previously, the vitamins were conjugated with polymers like retinoic acid with heparin [40] or co-loaded with tumor drugs for simultaneous delivery of retinoid acid and paclitaxel [41]. RET has been applied to initiate the ring-opening polymerization of lactide [42] and the controlled radical polymerization of methacrylates [43]. 4nBRE is used as a bifunctional derivative of vitamin A which has a positive effect on skin condition similar to monofunctional RET and has not been investigated as the polymer chain functionalizing moiety. Here we utilize modified 4nBRE, to a dibromoester derivative, for atom transfer radical polymerization (ATRP) to design V-shaped copolymers of methyl methacrylate (MMA), 2-hydroxyethyl methacrylate (HEMA) (P(HEMA–*co*–MMA)), and MMA or HEMA copolymers with poly(ethylene glycol) methyl ether methacrylate (MPEGMA) (P(MMA–*co*–MPEGMA) and P(HEMA–*co*–MPEGMA)). Moreover, another series of grafted copolymers was also obtained by using “click” chemistry reaction as “grafting onto” method. For this purpose, multifunctional copolymers with alkyne functionalized HEMA (AlHEMA) were obtained. Next, the P(AlHEMA–*co*–MMA)s were subjected to a “click” reaction with azide functionalized poly(ethylene glycol) (PEG-N_3_). The main structural characteristics of the copolymers were characterized by standard spectroscopy and chromatography methods. The V-shaped copolymers, both linear and graft, were able to self-assemble in aqueous solutions and also demonstrate the ability to encapsulate selected active substances (VitC, VitE, or ARB), these active substances have been shown to have anti-wrinkle and skin brightening properties by using a dialysis method. The micellization behavior of the obtained amphiphilic copolymers was studied using fluorescence spectroscopy, light scattering, and transmission electron microscopy. The release kinetics were monitored by UV transmittance. Additionally, the physicochemical aspect of the drug delivery was compared for two types of PEG graft copolymers, which were prepared by different strategies, one by ‘grafting through’ a macromonomer and the second by side chain ‘click’.

## 2. Materials and Methods 

### 2.1. Materials

Methyl methacrylate (MMA, 99%, Alfa Aesar, Warsaw, Poland), poly(ethylene glycol) methyl ether methacrylate (MPEGMA, Aldrich, *M*_n_ = 500 g/mol, 97%, Poznań, Poland), 2-hydroxyethyl methacrylate (HEMA, Aldrich, 97%, Poznań, Poland), methanol (Alfa Aesar, 99%, Warsaw, Poland) and anisole (Alfa Aesar, 99%, Warsaw, Poland) were dried over molecular sieves and stored in a freezer under nitrogen. Copper (I) bromide (CuBr, Fluka, 98%, Steinheim, Germany) was purified by previously reported procedure [43]. 4,4-Dinonyl-2,2-dipyridyl (dNdpy, Aldrich, 97%, Poznań, Poland), *N,N,N′,N″,N″*-pentamethyldiethylenetriamine (PMDETA, Aldrich, 98%, Poznań, Poland), triethylamine (TEA, Aldrich, 99%, Poznań, Poland), pyridine (Aldrich, 99%, Poznań, Poland), 2-bromoisobutyryl bromide (BriBuBr, Aldrich, 98%, Poznań, Poland), 5-hexynoic acid (HexA, Acros, 97%, Geel, Belgium), 4-*n*-butylbenzene-1,3-diol (4nBRE, Ark Pharm, 95%, Gdańsk, Poland), poly(ethylene glycol)methyl ether 2-bromoisobutyrate (PEG-Br, Aldrich, *M*_n_ = 1200 g/mol, Poznań, Poland), sodium azide (NaN_3_, Alfa Aesar, 99%, Karlsruhe, Germany), *N,N′*-dicyclohexylcarbodiimide (DCC, Acros, 99%, Geel, Belgium), 4-dimethylaminopyridin (DMAP, Acros, 99%, Geel, Belgium), *N,N*-dimethylformamide (DMF, 99%, Chempure, Piekary Śląskie, Poland), tetrahydrofurane (THF, Chempure, Piekary Śląskie, Poland), L(+)-ascorbic acid (VitC, Chempure, 99%, Piekary Śląskie, Poland), arbutin (ARB, Acros, 95%, Geel, Belgium), (±)-α-tocopherol (VitE, Acros, 96%, Geel, Belgium) and a 0.1 M sodium phosphate buffer solution (PBS; pH = 7.4, Aldrich, Poznań, Poland) were used as received.

### 2.2. Modification of HEMA to the Alkyne Derivative (2-(Prop-1-en-2-carbonyloxy)ethyl Hex-5-Ynate, AlHEMA)

Into a solution of HEMA (3.00 mL, 24.67 mmol) and DCC (5.67 g, 27.48 mmol) in methylene chloride (50 mL), hexynoic acid (2.80 g, 24.97 mmol) was dropwise added. After cooling to 0 °C, DMAP (0.1397 g, 1.14 mmol) in methylene chloride (2 mL) was added. The reaction was then carried out at room temperature for 2 days and then the mixture was extracted three times with methylene chloride/H_2_O. The brown liquid obtained after evaporation of the organic fraction was dried to constant mass [44]. Yield: 61%. ^1^H NMR (300 MHz, CDCl_3_, ppm): 6.14 and 5.61 (2H, =CH_2_), 4.35 (4H, –OCH_2_CH_2_O–), 2.52 (2H, –OC(=O)CH_2_–), 2.28 (2H, –CH_2_-C≡CH), 1.99 (1H, –C≡CH), 1.95 (3H, –CH_3_), 1.81 (2H, –OC(=O)CH_2_CH_2_–). ^13^C NMR (300 MHz, DMSO, ppm): 172 (C7, –OC(=O)CH_2_–), 166 (C4, –CC(=O)O), 136 (C2, CH_2_=C–), 126 (C1, CH_2_=C–), 83 (C11, –C≡CH), 72 (C12, –C≡CH), 63 (C5, –OCH_2_CH_2_O–), 62 (C6, –OCH_2_CH_2_O–), 32 (C8, –OC(=O)CH_2_–), 27 (C9, –OC(=O)CH_2_CH_2_–), 18 (C10, –CH_2_-C≡CH), 17 (C3, –CH_3_). Electrospray ionization (ESI) MS (m/z): calculated for C_12_H_16_O_4_, 224.0; found for [M + Na]^+^, 247.1.

### 2.3. Synthesis of 2-Bromoisobutyrate Derivative of 4-n-Butylbenzene-1,3-diol (4-Butyl-1,3-phenylene bis(2-bromo-2-methylpropanoate), 4nBREBr_2_)

A 1.0 g (6.02 mmol) of 4nBRE was solved in THF (40 mL), and TEA (1.85 mL, 13.27 mmol) was dropwise added. After cooling to 0 °C, BriBuBr (1.64 mL, 13.27 mmol) was dropped. The reaction was then carried out at room temperature for 1 day and then the precipitated was filtered. The resulting clear solution was evaporated and dried under vacuum to constant mass. Yield: 97%. ^1^H NMR (300 MHz, DMSO, ppm): 7.18 (1H, –CH=, aromat.), 7.04 (1H, –CH=, aromat.), 7.02 (1H, –CH=, aromat.), 2.60 (2H, –CH_2_–, aliphat.), 2.16 (12H, 2* –C(CH_3_)_2_Br), 1.59 (2H, –CH_2_–, aliphat.), 1.38 (2H, –CH_2_–, aliphat.), 0.98 (3H, –CH_3_, aliphat.). ^13^C NMR (400 MHz, DMSO, ppm) δ: 174 (C11, –OC(=O)–), 153 (C1, –CH=, aromat.), 149 (C3, –CH=, aromat.), 129 (C5, –CH=, aromat.), 128 (C4, –CH=, aromat.), 117 (C6, –CH=, aromat.), 114 (C2, –CH=, aromat.), 65 (C13, –OC(=O)**C**–), 42 (C7, C8, –CH_2_–), 36 (C12, –CH_3_), 29 (C9, –CH_2_–), 18 (C10, –CH_3_). ESI-MS (m/z): calculated for C_18_H_24_Br_2_O_4_ 462.0; found for [M + Na]^+^ 486.0 (Figure S1, Supplementary Materials).

### 2.4. Synthesis of P(HEMA-co-MMA) (Example for I) 

In a Schlenk flask, HEMA (1.60 mL, 13.16 mmol), MMA (4.23 mL, 39.55 mmol), anisole (0.82 mL, 10 vol % of monomer), dNbpy (53.82 mg, 0.132 mmol) and 4nBREBr_2_ (65.10 mg, 0.132 mmol) were mixed. Next, three freeze–pump–thaw cycles were applied to degas the reaction mixture, and a catalyst (CuBr, 18.88 mg, 0.132 mmol) was added. The reaction was then carried out at 60 °C (in an oil bath) and stopped by exposure to air. CuBr was removed from reaction mixture by cationite (Dowex). The product was precipitated in diethyl ether, isolated by decantation and dried to constant mass.

### 2.5. Synthesis of P(HEMA-co-MPEGMA) (Example for VIII)

In a Schlenk flask, MPEGMA (4.97 mL, 10.74 mmol), HEMA (3.92 mL, 32.23 mmol), dNbpy (98.65 mg, 0.241 mmol), 4nBREBr_2_ (53.00 mg, 0.107 mmol), and solvents (0.89 mL, 10 vol % of monomers, methanol:anisole = 1:9 (*v*/*v*)) were mixed. Next, three freeze–pump–thaw cycles were applied to degas the reaction mixture, and a catalyst (CuBr, 15.39 mg, 0.107 mmol) was added. The reaction was then carried out at 60 °C (in an oil bath) and stopped by exposure to air. After dissolving the reaction mixture in chloroform, CuBr was removed by cationite (Dowex). The product was precipitated in heptan, isolated by decantation, and dried to constant mass.

### 2.6. Synthesis of P(MMA-co-MPEGMA) (Example for X)

In a Schlenk flask, MPEGMA (5.43 mL, 11.73 mmol), MMA (3.77 mL, 35.24 mmol), dNbpy (107.96 mg, 0.264 mmol), 4nBREBr_2_ (58.00 mg, 0.117 mmol), and solvents (0.92 mL, 10 vol % of monomers, methanol:anisole = 1:9 (*v*/*v*)) were mixed. Next, three freeze–pump–thaw cycles were applied to degas the reaction mixture, and a catalyst (CuBr, 16.84 mg, 0.117 mmol) was added. The reaction was then carried out at 60 °C (in an oil bath). Further procedure was the same as in the previously described P(HEMA–*co*–MPEGMA) synthesis (Section 2.5).

### 2.7. Synthesis of P(AlHEMA–co–MMA) (Example for IV)

In a Schlenk flask, MMA (2.90 mL, 27.11 mmol), AlHEMA (2.04 g, 9.11 mmol), dNbpy (74.62 mg, 0.183 mmol), 4nBREBr_2_ (45.10 mg, 0.091 mmol), and anisole (0.49 mL, 10 vol % of monomers) were mixed. Next, three freeze–pump–thaw cycles were applied to degas the reaction mixture, and a catalyst (CuBr, 13.09 mg, 0.091 mmol) was added. The reaction was carried out at 60 °C (in an oil bath) and stopped by exposure to air. After dissolving the reaction mixture in acetone, CuBr was removed by passing the mixture through a neutral alumina column. The product was precipitated in diethyl ether, isolated by decantation, and dried to constant mass.

### 2.8. Modification to an Azido Derivative of Poly(Ethylene Glycol) Monomethyl Ether (PEG-N_3_) 

A solution of PEG-Br (1 g, 0.83 mmol) and NaN_3_ (54.16 mg, 0.83 mmol) in anhydrous DMF (20 mL) was stirred for one day at room temperature. Then, the reaction mixture was extracted three times with CH_2_Cl_2_/NaHCO_3(aq)_ and the obtained organic phase was concentrated. The product (brown liquid) was dried to a constant mass [40]. Yield: 88%. ^1^H NMR (300 MHz, DMSO, ppm): 3.50 (n*4H, –[OCH_2_CH_2_]*_n_*–), 3.24 (3H, –OCH_3_), 1.88 (6H, –C(CH_3_)_2_N_3_). ^13^C NMR (300 MHz, DMSO, ppm): 167 (C4, –OC(=O)–), 75 (C2 and C3, –OCH_2_CH_2_O–), 65 (C1, –OCH_3_), 60 (C5, –C(CH_3_)_2_N_3_), 41 (C6, –C(CH_3_)_2_N_3_).

### 2.9. “Click” Chemistry Azide-Alkyne Reaction (Example for IVc)

To a solution of polymer IV (0.20 g, 7.955 × 10^−3^ mmol containing 0.37 mmol of AlHEMA units) in DMF (10 mL), PEG-N_3_ (the equimolar amount: 0.49 g, 0.37 mmol) and PMDETA (2.5-fold molar excess: 0.20 mL, 0.93 mmol) were added. After purification of the reaction mixture (20 min, inert gas), CuBr (0.13 g, 0.93 mmol) was added and the reaction was completed for 2 days maintaining a constant mixing (r.t.; without access to light). CuBr was removed by cationite (Dowex) and the obtained solution was concentrated. The product was precipitated in diethyl ether and dried to constant mass.

### 2.10. Loading of the Active Substance into Polymeric Micelles

To a solution of the amphiphilic copolymer (100 mg) and active substance (the weight ratio of copolymer: active substance = 1:1) in methanol (15 mL), H_2_O was added dropwise (200 vol % of the solvent) ensuring constant mixing. The encapsulation process took place over a 24-h period, then the organic solvent was evaporated and the unloaded active substance was separated by centrifugation (4000 rpm for 10 min, r.t.). The purified aqueous fraction was lyophilized by freezing to obtain a solid product. A solution of loaded micelles in MeOH (0.008 mg/mL) was prepared to determine the amount of entrapped substances by using ultraviolet-visible light spectroscopy (UV–Vis, Thermo Fisher Scientific Evolution 300, resolution >2.0 at 0.5 nm SBW), measuring the absorbance at λ = 282 nm for ARB, λ = 267 nm for VitC and λ = 298 nm for VitE. Each measurement was performed three times at room temperature and the results obtained were averaged. Drug loading content (DLC) was calculated using the following equation

(1) $$DLC=\;\frac{\mathrm{Weight}\;\mathrm{of}\;\mathrm{drug}\;\mathrm{loaded}\;\mathrm{into}\;\mathrm{micelle}}{\mathrm{Weight}\;\mathrm{of}\;\mathrm{total}\;\mathrm{polymer}\;\mathrm{and}\;\mathrm{loaded}\;\mathrm{drug}}\times 100\%$$

### 2.11. Active Substance Release Studies

The solution of loaded micelles in PBS (pH = 7.4, 1.0 mg/mL) was placed into a dialysis membrane bag (cellulose, MWCO = 3.5 kDa) and incorporated into vial with PBS (50 mL). The release process was performed in a water bath (37 °C) ensuring constant mixing. To calculate the concentration of the released drug, the absorbance of the released medium samples was measured by UV–Vis spectroscopy. The absorbance of active substances was determined experimentally: λ = 282 nm for ARB, λ = 267 nm for VitC, and λ = 298 nm for VitE. In the first hour of the experiment, samples were taken every 10 min and then every 30 min after this until release was completed.

### 2.12. Characterization

^1^H and ^13^C NMR spectra were recorded with a UNITY/INOVA (Varian, Mulgrave, Victoria, Australia) spectrometer operating at 300 MHz and 75 MHz, respectively, using dimethyl sulfoxide (DMSO) or chloroform (CDCl_3_) as solvents and tetramethylsilane (TMS) as an internal standard (*δ*_iso_(^1^H,^13^C) = 0 ppm). The monomer conversion was determined by gas chromatography (GC, Agilent Technologies 6850 Network GC System, Santa Clara, CA, USA), which was equipped with a flame ionization detector. The measurements were completed in acetone and optimizing the temperature of the injector and detector to 250 °C, initial and final temperature of column 40 and 200 °C, respectively. The integration of signals at defined retention times for MPEGMA (1.8 min), MMA (2.3 min), HEMA (8.5 min), and AlHEMA (10.0 min) were compared to the retention time of anisole (4.9 min), allowing us to calculate monomer conversion [43,44]. Mass spectrometry (MS, Xevo G2 QTof, Waters Corporation, Milford, UT, USA) was used to confirm the molecular masses of the modified 4nBRE and functionalized HEMA. Molecular weights (*M*_n_) and dispersity indices (*Đ*) were determined by gel permeation chromatography (GPC, 1100 Agilent 1260 Infinity, Santa Clara, CA, USA) equipped with an isocratic pump, autosampler, degasser, thermostatic box for columns, and differential refractometer MDS RI Detector. The measurements were carried out in a tetrahydrofuran (THF) solvent at 30 °C with a flow rate of 0.8 mL/min. The GPC calculations were calibrated with the use of linear polystyrene standards (580–300,000 g/mol). Fourier-transform infrared spectroscopy (FT-IR) was conducted with a Perkin-Elmer Spectrum Two 1000 FT-IR Infrared Spectrometer (Perkin Elmer, Waltham, MA, USA) using attenuated total reflection (ATR). Spectra were recorded at 32 scans per spectrum and 4 cm^−1^ resolution over a range of 4000–400 cm^−1^. The critical micelle concentration (CMC) was measured by fluorescence spectrophotometry (FL, fluoroSENS Pro-11 spectrofluorimeter, CAMLIN, Lisburn, Ireland), using pyrene as a fluorescence probe. Excitation spectra of pyrene (λ = 390 nm) were recorded at a constant concentration (3.0 × 10^−4^ mol/L) and polymer concentrations in the range of 5 × 10^−4^ to 1.0 mg/mL. The intensity ratio (I_336_/I_332_) from the pyrene excitation spectrum vs. logC (where C is the concentration in mg/mL), where the cross-over point was determined to be a CMC value [17,18]. The particle sizes and their distributions, that is, hydrodynamic diameter (*D*_h_) and polydispersity index (PDI) were measured at 25 °C using dynamic light scattering (DLS, Zetasizer Nano-S90, Zetasizer Software, Malvern Technologies, Malvern, UK) equipped with a He-Ne laser at a fixed scattering angle (173°). Samples were measured in PMMA cells and size measurements were carried out on two samples from three independent runs to obtain an average value. The samples taken during the release process were analyzed by ultraviolet-visible light spectroscopy (UV–Vis, Thermo Fisher Scientific Evolution 300, Waltham, MA, USA) to determine the DLC and the amount of released substance over time. The measurements were carried out in poly(methyl methacrylate) cells. The surface morphology of the micelles was observed by scanning electron microscopy (SEM) using a Phenom X Pro electron microscope (Phenom-World Bv, Eindhoven, The Netherlands). Before measurements, the samples were dusted with a layer of 5 nm gold nanoparticles. For transmission electron microscopy (TEM), the samples were prepared by adding 3 µL of the polymer solution, at a concentration of 1.0 mg/mL, onto glow discharged copper grids coated with carbon films and left at room temperature to dry. Grids were analyzed using an FEI Tecnai G2 Spirit BioTwin TEM (Eindhoven, The Netherlands) at 120 kV.

## 3. Results

**Figure 1:**
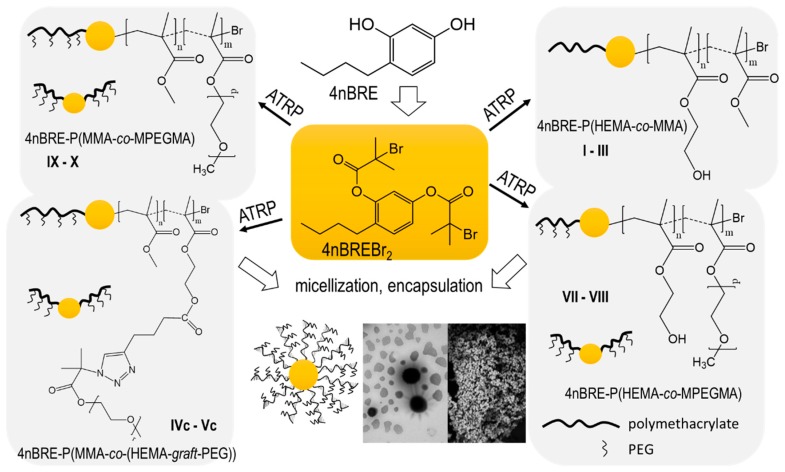
Amphiphilic linear and graft copolymers prepared with 4nBREBr_2_ as initiator.

Four series of copolymers were synthesized by pseudo-living ATRP, which was initiated by bromoester-functionalized 4-*n*-buthylresorcinol (4nBREBr_2_) and catalyzed with CuBr/dNbpy in anisole or anisole/methanol at 60 °C. Various initial ratios (25/75, 50/50, and 75/25) of the methacrylate comonomer pairs (HEMA/MMA, AlHEMA/MMA, HEMA/MPEGMA, and MMA/MPEGMA) were applied to vary the hydrophilic/hydrophobic ratios of the copolymers. Additionally, the hydrophobic P(AlHEMA–*co*–MMA)s were subjected to the Cu(I) catalyzed 1,3-dipolar azide-alkyne cycloaddition (CuAAC) to attach hydrophilic PEG side-chains. This procedure is presented in Figure 1, which includes the preparation of the initiator and its use in copolymerization, the ‘click’ chemistry reaction between P(AlHEMA–*co*–MMA), and PEG-N_3_ and the self-assembling of amphiphilic copolymers into micellar structures.

**Figure 2:**
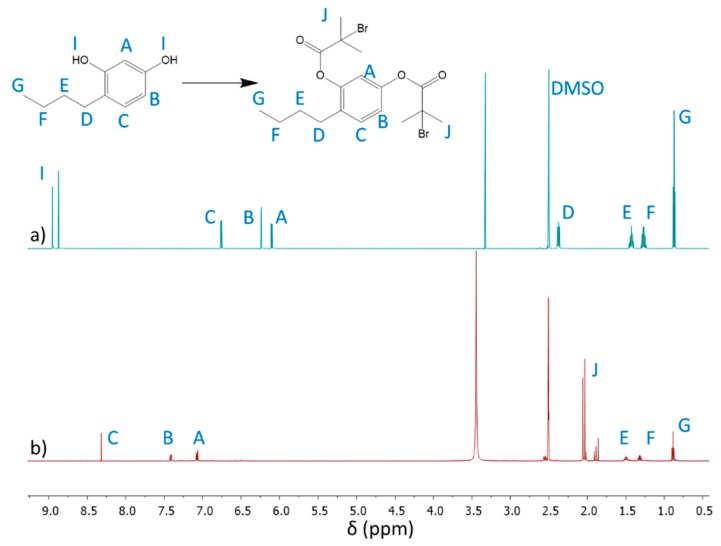
^1^H NMR spectra of (**a**) 4nBRE, and (**b**) after its modification to initiator 4nBREBr_2_.

The 4nBREBr_2_ as dibromoester derivate of 4nBRE was achieved by using an esterification reaction between the OH groups of 4nBRE and BriBuBr. The structure of 4nBREBr_2_ was elucidated by ^1^H NMR, showing the disappearance of hydroxyl group signals **I** (8.87 and 8.94 ppm) and the appearance of a new signal **J** (2.16 ppm), representing methyl protons at the bromide site after esterification (Figure 2). Due to changes in the local electronic structure following the formation of the ester groups, the aromatic ring protons shift (Figure 2a: A: 6.11 ppm, B: 6.24 ppm, C: 6.75 ppm vs. Figure 2b: A: 7.08 ppm, B: 7.41 ppm, C: 8.31 ppm). The successful modification was also confirmed by ^13^C NMR (Figure S2 in Supplementary Materials) showing the formation of ester resonances –O**C**(=O)– (C11: 174 ppm), tertiary carbons bonded to bromide –OC(=O)C– (C13: 65 ppm), and carbons from methyl groups in the vicinity of the ester group –CH_3_ (C12: 36 ppm). 

**Figure 3:**
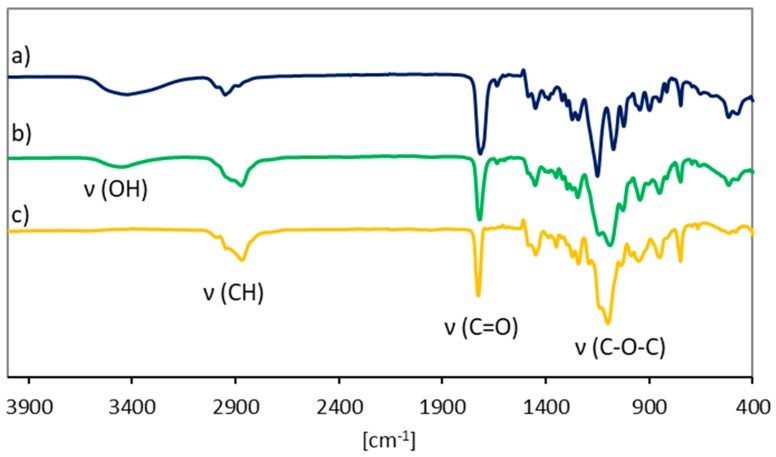
FT-IR spectra for copolymers (**a**) P(HEMA–*co*–MMA) **III**, (**b**) P(HEMA–*co*–MPEGMA) **VIII** and (**c**) P(MMA–*co*–MPEGMA) **IX**.

The prepared bifunctional initiator 4nBREBr_2_ was used in the copolymerization of the different monomers (MMA, HEMA, AlHEMA, MPEGMA), in the presence of a CuBr/dNbpy catalyst system in anisole or anisole/methanol solutions at 60 °C (Table 1). Due to the presence of two bromoester initiating groups, the obtained copolymers are assumed to be V-shaped, as it has been previously reported for polymethacrylates with methyl 4,6-*O*-benzylidene-α-d-glucopyranoside core [45]. The presence of a bioactive 4nBRE moiety in the center of the polymeric chain can improve its biocompatibility as it has been observed for dextran linking *trans*-ferulic acid [46]. In comparison to the HEMA/MMA (I-III) and AlHEMA/MMA (IV-VI) linear copolymers, the grafted copolymers were synthesized with the use an MPEGMA macromonomer, which significantly increases the hydrophilicity of the systems (VII-X) due to the PEG side chains [47]. Moreover, the polymerizations carried out with MPEGMA showed high comonomer conversions of above 60% (Table 1). The degree of hydrophilicity was controlled by an appropriate selection of the initial proportions of the MMA or HEMA and MPEGMA comonomers (50/50 and 75/25). The presence of hydroxyl groups in the P(HEMA–*co*–MMA) and P(HEMA–*co*–MPEGMA) copolymers is observed in the FT-IR spectra (Figure 3a,b), given as broad peak at 3200–3500 cm^−1^ in contrast to the copolymer without HEMA units such as P(MMA–*co*–MPEGMA) (Figure 3c), which is in agreement with analogous polymethacrylates [43,44]. MPEGMA containing copolymers exhibited a high intensity extended band in the range of 1050–1150 cm^−1^ representing C–O–C stretching vibrations (Figure 3b,c).

**Figure 4:**
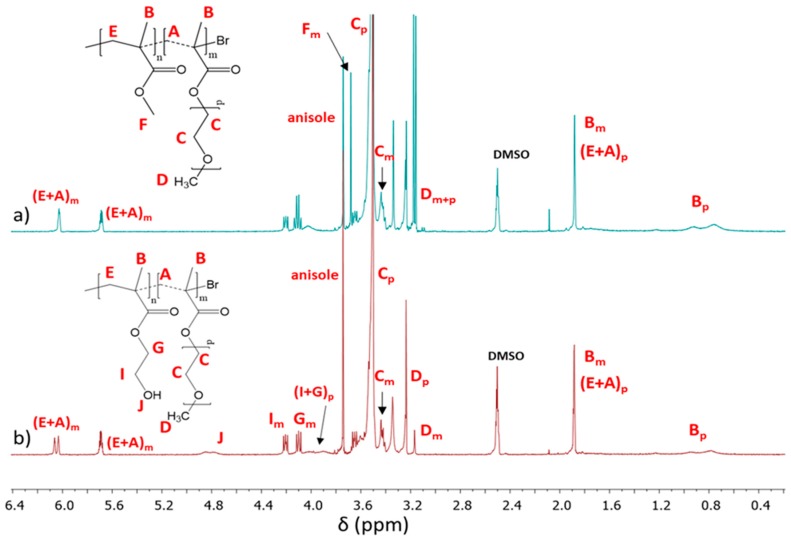
^1^H NMR spectra of reaction mixture for 4nBREBr_2_ initiated copolymerization of (**a**) MMA/MPEGMA: 50/50 (**IX**) and (**b**) HEMA/MPEGMA: 50/50 (**VII**), where signals with indices m and p are related to monomer and polymer, respectively.

**Figure 5:**
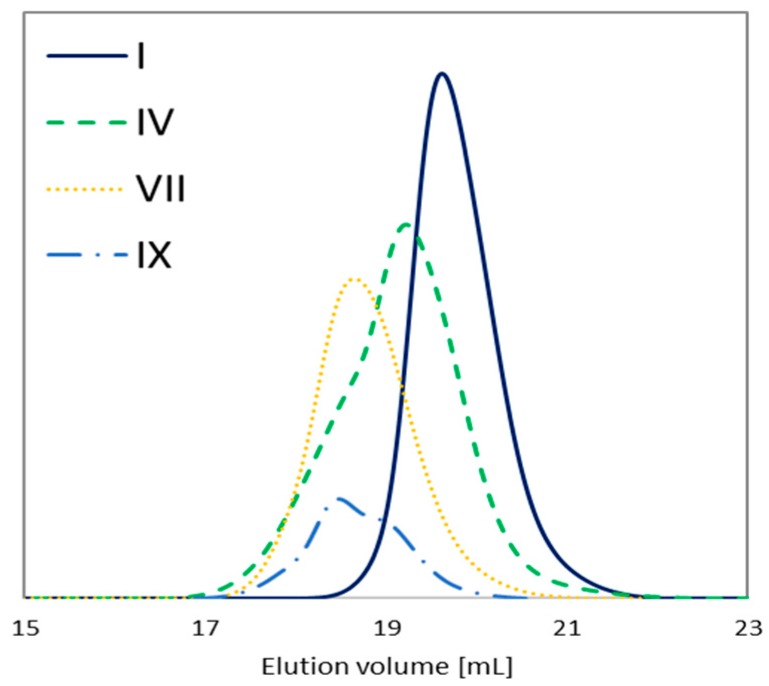
GPC traces of representative copolymers.

The conversion of the monomers into copolymers was calculated from the ^1^H NMR spectrum by integrating the resonances related to unreacted monomer and formed polymer. P(AlHEMA–*co*–MMA) and P(HEMA–*co*–MMA) protons which represented methylene groups (CH_2_=_mon_: 5.6 and 6.2 ppm; –COO–CH_2_-_polym_: 4.1–4.2 ppm) and protons in methoxy groups (–OCH_3mon_: 3.75 ppm, –OCH_3polym_: 3.6 ppm) were used to evaluate the conversion of AlHEMA or HEMA and MMA, respectively [44]. For other copolymers the MPEGMA conversion was determined via integration of isolated resonances assigned to CH_2_= in the monomer (A_m_: 6.03 ppm) and –OCH_2_CH_2_O– in the polymer (C_p_: 3.52 ppm) [48] (Figure 4). The integrals of monomer conversion by ^1^H NMR coincided with those calculated from GC analysis, which gives well-separated signals and, therefore, for all further calculations of molecular weight (*M*_n_) and DP, the conversion by GC was used as the method to determine conversion to reduce propagation of errors. The chains of P(AlHEMA–*co*–MMA)s obtained with the use of bifunctional 4nBREBr_2_ (DP = 120–225) are typically longer in comparison to those previously synthesized with two different ATRP initiators, the standard ethyl 2-bromoisobutyrate and RET based bioinitiator [43] with an initiating group (Figure S3 in Supplementary Materials). Comparable monomer conversions (HEMA vs. MMA and AlHEMA vs. MMA) suggests the formation of statistical copolymers, whilst for HEMA vs. MPEGMA and MMA vs. MPEGMA significantly lower conversion of the macromonomer (especially at a ratio of 75/25) is responsible for the gradient structure of these copolymers. Almost all copolymers showed relatively low dispersity indices with symmetrical signals of the GPC traces (Table 1, Figure 5), which confirmed the controlled growth of the polymeric chains. Although in some cases the occurrence of side reactions caused a slight broadening of the signal due to the presence of alkyne groups (IV) or bimodal signals because of the specific hindrance of the macromonomer (IX), their influence on the changes of physicochemical properties of polymers can be omitted.

Furthermore, the AlHEMA/MMA copolymers (IV-VI) were grafted by PEG chains using a Huisgen “click” chemistry CuAAC reaction catalyzed by CuBr/PMDTA in DMF the between azide and alkyne moieties forming 1,4-substituted triazole rings. This creates a grafted copolymer P((HEMA–*graft*–PEG)–*co*–MMA) with amphiphilic properties and the ability to undergo micellization and encapsulate active substances. The “click” reactions were carried out with high efficiencies (75–80%) (Table 2), when compared to previously synthesized analogous graft copolymers (containing ethyl isobutyrate or RET starting group) [40] with “click” yields of 20–65% (Figure S4 in Supplementary Materials).

Variety of copolymer composition based on the nature and proportion of comonomers has influence on its hydrophilic–hydrophobic balance. The critical micelle concentration (CMC, Table 3) was determined to evaluate the self-assembling behaviors of the graft copolymers. CMC values of copolymers were measured using a standard procedure to present the semilogarithmic plot of I_336_/I_332_ vs. the concentration of the copolymer (Figure S5 in Supplementary Materials). In most cases, it was noted that the CMC increased with the content of the hydrophilic fraction within the series (I–III, IVc vs. Vc and VII–VIII). The highest CMC is observed for the graft copolymers by “grafting onto”, e.g., P((HEMA–*graft*–PEG)–*co*–MMA), whereas the highest CMC for the copolymers by “grafting through” was twice lower as that observed for P(HEMA–*co*–MPEGMA) with a content of 40% of the hydrophilic fraction. The hydrodynamic diameters (D_h_) of the formed self-assembling particles were determined by DLS in aqueous solution (Table S2 in Supplementary Materials). The micellization resulted in polymeric systems with variable characteristics, with one superaggregate fraction for the less hydrophilic linear copolymer (I), two fractions for the unimers, micelles for the “click” grafted copolymers and MPEGMA copolymer with significant hydrophobic domination (IVc, Vc, X), and three fractions for the more hydrophilic linear copolymers and graft copolymers containing more than 40% MPEGMA (II, III, VII, IX) (Table S2, Figures S6 and S7 in Supplementary Materials). 

**Figure 6:**
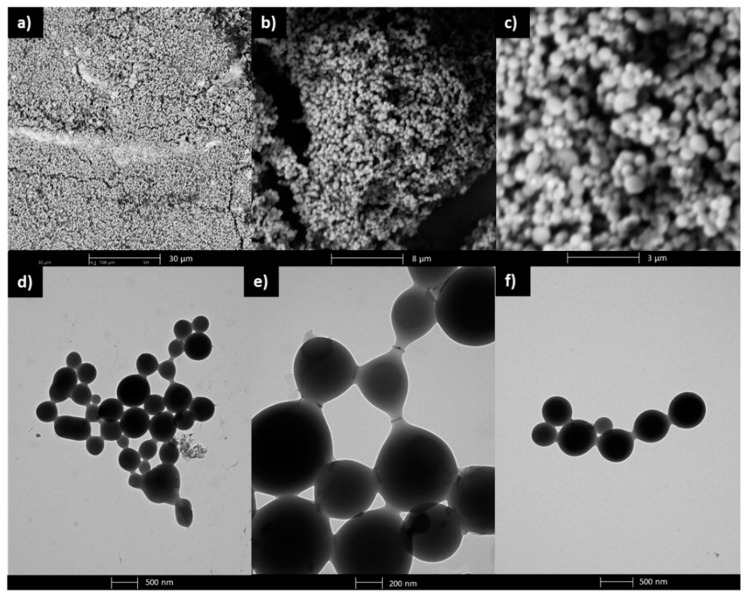
SEM images (**a***–***c**) and TEM micrographs (**d***–***f**) for empty micelles formed by copolymer I, where a photo (**e**) is magnification of a photo (**d**).

The empty micelles formed by linear P(HEMA–*co*–MMA) (I) were subjected to SEM and TEM analyses to observe the surface morphology and ultrastructural qualities. The SEM images confirmed the formation of spherical micellar superstructures which aggregate and form a layered structure (Figure 6a–c). The hydrodynamic diameters of the individual micelles measured by SEM reached sizes of 380–640 nm. The micellar nature illustrated on the TEM micrographs reveal a smooth surface and uniform internal structure of the nanoparticles (Figure 6d–f). Additionally, the spherical micelles stick together through elongated bridges creating specific art shape aggregates, including nanochain architecture. Similarly, the intermicellar linear aggregation developed by random collisions and containing strong interactions between the spherical nano-objects has been reported for PEGMA graft copolymers containing cholesteryl moieties (230–460 nm) [49] and block copolymers containing poly(benzyl methacrylate) and PEGMA based segments (100–600 nm) [50].

**Figure 7:**
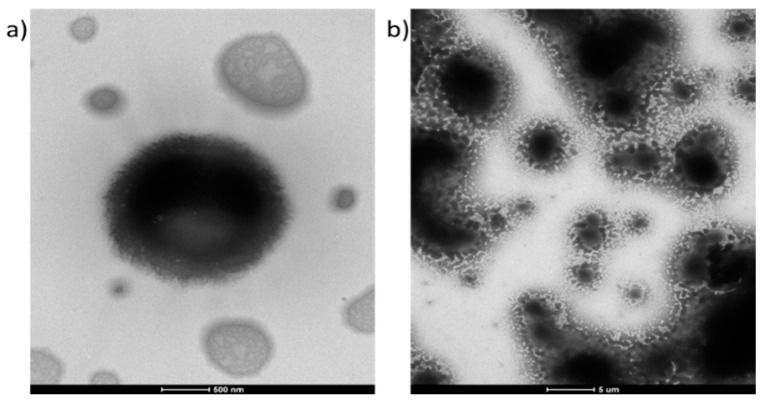
TEM images of self-assembling II (**a**) and IVc (**b**) copolymers.

The morphology of the micelles formed by polymers II and IVc are presented in Figure 7. For the P(HEMA–*co*–MMA) copolymer with an equal content of both types of repeating units (Figure 7a, Figure S8a in Supplementary Materials) at least two morphological forms are observed, one shows micelles with complex/fold surface, and the second is a smaller, flat, and dried polymer, which has a complex internal structure and a visible electron homogenous layer around. It is known that during the drying processes, polymeric assemblies respond differently to this phenomenon [49]. Thus, for this case, the micelles were disrupted from micellar assembly into a singular or aggregated flat irregular structures described above during the drying process. The TEM images of the ‘click’ grafted polymer IVc (Figure 7b, Figures S8b–f and S9, Supplementary Materials) show different morphology for a film with varying thickness, that is, a continuous grainy layer visible in the thinnest part of the sample with a tendency to have increasing granularity (Figure S8b,c), and a continuous film with a maze of crevices like a sponge structure in the thicker areas (Figure S8d–f, Supplementary Materials). The presence of a continuous layer at the top of the thicker film (Figure S8e, Supplementary Materials) and micrometre-range size particles were visible in these samples (Figure S9 in Supplementary Materials).

Previously, satisfactory encapsulation of VitC and ARB by linear or graft copolymers functionalized with RET had been observed [43] and this encouraged us to prepare the micellar systems based on the 4nBRE-functionalized copolymers in the presence of VitE, VitC, or ARB. However, the studies showed that in some cases it was not possible to encapsulate and release effectively these substances using synthesized copolymers (Table S1 in Supplementary Materials). In contrast to RET based P(HEMA–*co*–MMA), the encapsulation of VitC was inefficient by copolymers with 4nBRE as the starting group, both linear (I–III) and the “click” grafted series (IVc, VIc), yielding just a 1% loading. Fortunately, these polymers encapsulated VitE and ARB. An inverse relationship was observed for the graft copolymers with MPEGMA units (VII–X), these demonstrated a good ability to form micelles with encapsulated VitC and VitE (10–50%), but in the latter case, the active substance was not released.

The encapsulation efficiency (polymer:drug = 1:1) was characterized using the drug loading content (DLC) from UV–Vis spectroscopy (Table 4). Micelles formed by linear copolymers I-II (VitE, 13–20%) and the highly hydrophilic HEMA/MPEGMA graft copolymer VII (VitC, 11%) show the lowest encapsulation efficiencies. High DLC values are obtained for micellar systems with “click” grafted copolymers with ARB (>50%) and MMA/MPEGMA graft copolymers with VitC (close to 50%), which maintained optimal sizes below 200 nm.

The loading of the biological active substance (Table 4) indicates that the particle sizes increase with the hydrophilic fraction in these polymers, but almost all the copolymers formed micelles with a D_h_ up to 200 nm, with exception of II which are slightly larger (∼225 nm). Similar trends have been observed in the amphiphilic RET based copolymers of HEMA/MMA [43]. The histograms in Figure S10 show two fractions of superstructures with significant predomination of one of them (94–95%) in the linear copolymer II, graft copolymers IVc, Vc, and a copolymer built almost entirely from hydrophilic units (VII). These systems do not support aggregation and superaggregation, demonstrating only unimers and micelles, with exception of sample II which yeilds 6% aggregates (∼450 nm). The small micelles with sizes of ∼100 nm were formed by the copolymers P(MMA–*co*–MPEGMA) (IX, X), but their amount was slightly lower (∼80%) due to the presence of two other fractions of particles, the unimers and superaggregates. A major fraction of the particles measured by volume was defined by their smaller sizes than in the case of intensity measurements by DLS (Figure S11 in Supplementary Materials). This effect can be explained by the better light scattering of larger particles than for the smaller ones [45]. Our earlier studies have shown a strong influence of the chemical nature of starting unit in the polymer chain (introduced by various initiators, such as bromide derivatives of ethyl isobutyrate vs. RET) on the interactions between the encapsulated bioactive substance and the copolymer [43]. This resulted in the RET functionalized copolymers forming smaller micelles than those without RET moieties. Here, the investigated ARB loaded micelles of the “click” grafted copolymer with 4nBRE moiety localized in the middle of V-shaped chains (Vc) show a similar level of a hydrophilic fraction [44] and have smaller D_h_, probably due to non-linear topology, than previously studied analogous system with of ARB and RET functionalized copolymer.

**Figure 8:**
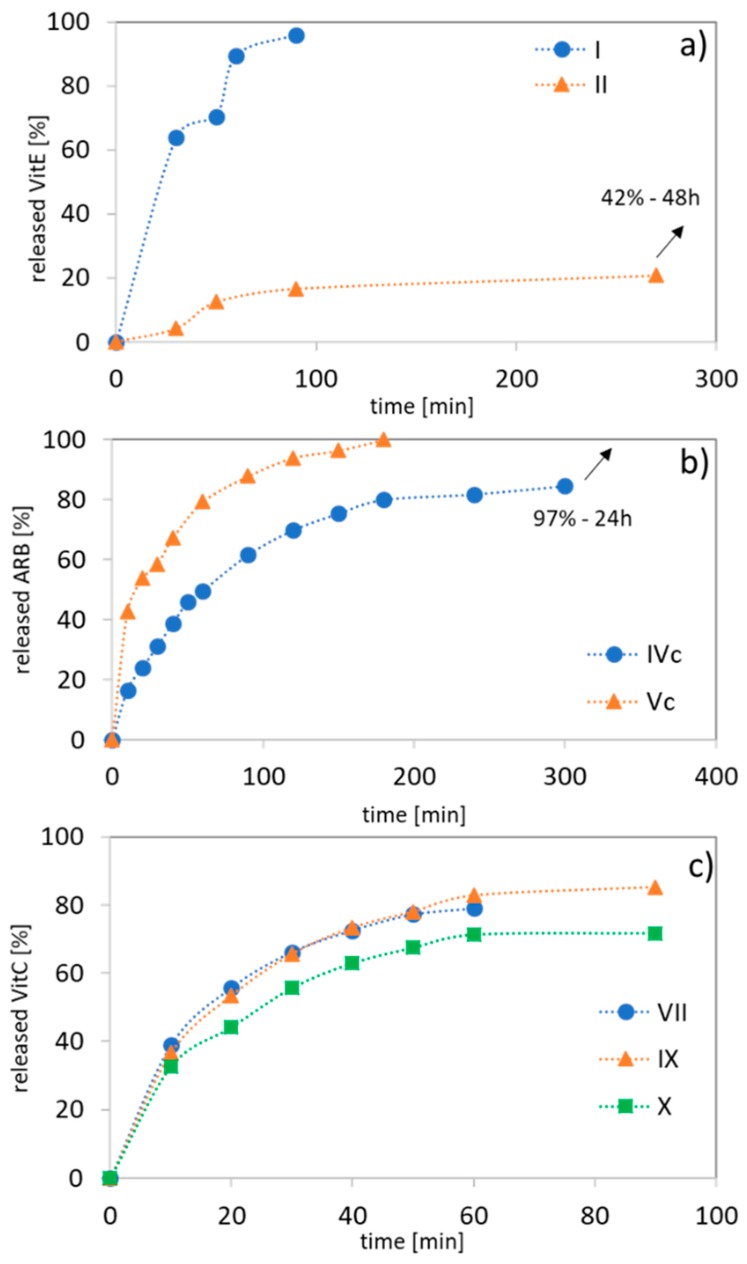
Kinetics profiles of release in PBS at pH 7.4 from self-assembling linear copolymers (**a**,**c**) and graft copolymers (**b**).

The kinetic profiles of ARB, VitC, or VitE (PBS, pH 7.4) show a range of release rates of the bioactive substance because of their differing hydrophilic contents, the drug’s nature, and the composition and topology of the carriers. For the ARB systems (IVc vs. Vc), the release was completed within the first 3 h due to the hydrophilic nature of the polymer, but it was significantly extended to 24 h with an increasing hydrophobic content (Table 4, Figure 8b). For VitE, which was encapsulated with the lowest amount, an inverse release correlation was observed for the linear MMA/HEMA copolymers completing the processes with the 1.5 h, in more hydrophobic systems (Figure 8a). A short release is also observed for MPEGMA copolymers which are independent of their composition (HEMA vs. MMA), where 70–85% of the maximum amount of VitC was released after 1–1.5 h (Figure 8c). Generally, fast release of vitamins is especially beneficial for cosmetic applications of the prepared systems as it was also stated for dextranes delivering vitamin E [46].

## 4. Conclusions

Bromoester functionalized 4-*n*-butylresorcinol was obtained and used as a novel bioinitiator with Cu-mediated ATRP to prepare copolymers of HEMA/MMA (linear), HEMA/MMA/PEG (“click” grafted), and MPEGMA (graft) with various hydrophilic/hydrophobic properties supporting the self-assembly behavior, in aqueous solution at room temperature. This has been achieved such that the standard ATRP initiator can be replaced with a suitably modified biologically active compound, which in addition improves the biocompatibility of the resulted polymers. All the obtained amphiphilic copolymers show the ability to form micelles, which is indicated by their CMC values and visualized by SEM and TEM. Most of the micellar systems, those based on graft copolymers, demonstrate satisfactory efficiencies of cosmetic substance encapsulation (50–73% of VitC and ARB), and almost complete in-vitro release within 60 min (up to a maximum of 90 min) with MPEGMA graft systems and 3 h (up to a maximum of 24 h) in the “click” grafted systems. The release tests allow us to conclude that copolymers with 4nBRE starter groups are promising micellar carriers for applications in cosmetology. The most representative systems will be selected for verification using specialized biological methods, such as transdermal tests in the Franz chambers and cytotoxicity, to confirm the possibility of using the obtained carriers in cosmetic products (wraps, creams, and masks).

## Figures and Tables

**Table 1:** Data for copolymers synthesized by ATRP initiated with 4nBREBr_2_.

	M_1_/M_2_	Time (h)	Monomer Conversion (%)				*DP*_n_^a^	*M*_n_^a^ (g/mol)	*M*_n_^b^ (g/mol)	*D* ^b^
			X_NMR_		X_GC_					
			M_1_	M_2_	M_1_	M_2_				
**I**	25/75	5.0	40	36	41	35	145	16,200	13,200	1.22
**II**	50/50	6.0	39	37	39	35	148	17,600	14,700	1.31
**III**	75/25	5.5	37	58	38	36	150	18,900	4000	1.75
**IV**	25/75	4.5	41	44	47	47	188	25,100	22,100	1.59
**V**	50/50	5.0	50	52	55	58	226	36,700	33,900	2.34
**VI**	75/25	5.5	26	30	29	33	118	22,900	17,800	1.51
**VII**	50/50	4.5	54	39	45	39	168	51,000	32,400	1.37
**VIII**	75/25	2.0	65	20	65	29	224	40,200	45,200	1.91
**IX**	50/50	4.5	84	40	61	44	210	56,300	53,700	1.40
**X**	75/25	3.5	62	31	51	32	186	31,800	37,300	1.31

Conditions: [M_1_ + M_2_]_0_/[4nBREBr_2_]_0_/[CuBr]_0_/[dNdpy]_0_, where I–III: HEMA/MMA, 400/1/0.75/1.5; IV–VI: AlHEMA/MMA, 400/1/1/2; VII–VIII: HEMA/MPEGMA, 400/1/1/2.25; IX–X: MMA/MPEGMA, 400/1/1/2.25; I–VI anisole 10 vol % of mon., VII–X anisole/methanol = 9:1 20 vol % mon; 60 °C; ^a^ calculated with the use of conversion by GC analysis; ^b^ determined by GPC.

**Table 2:** Characteristics of “click” graft copolymers P((HEMA–*graft*–PEG)–*co*–MMA).

	*DP*_AlHEMA_	*F*_AlHEMA_ (%)	*E*_click_ (%)	*n*_triazole_	*DG* (%)	*M*_n,NMR_ (g/mol)
IVc	47	25	74	35	19	34,600
Vc	110	49	81	89	39	60,900

DP_AlHEMA_—polymerization degree of AlHEMA; F_AlHEMA_—content of hydrophilic fraction in the copolymer; E_click_—efficiency of ‘click’ reaction calculated by integral area of signals from the CH proton in the triazole ring and the ≡CH proton from not clicked AlHEMA units; *n*_triazole_—number of triazole ring in the copolymer; DG—degree of grafting.

**Table 3:** CMC characteristics of self-assemblies.

	F_hydrophil_ (mol %)	CMC (mg/mL)
**I**	28	0.0017/0.0057
**II**	53	0.0140
**III**	76	0.0252
**IVc**	19	0.2705
**Vc**	39	0.3414
**VII**	46 *	0.1634
**VIII**	13 *	0.1063
**IX**	42	0.0071
**X**	17	0.0194

F_hydrophil_—content of hydrophilic fraction, * due to the presence of two hydrophilic units, the content of MPEGMA is given.

**Table 4:** Encapsulation and release of active substances.

	F_hydrophil_ (mol %)	Active Substance	DLC (%)	D_h_^a,b^ (nm)			PDI	Released Active Substance (%)	Time (h)
				Intensity	Volume				
**I**	28	VitE	13	95^c^		28	0.480	96	1.5
**II**	53		20	223		218 ^c^	0.233	42	48
**IVc**	19	ARB	73	148		23	0.246	97	24
**Vc**	39		55	174		21	0.266	100	3
**VII**	46 *	VitC	11	175		10	0.555	79	1.0
**IX**	42		47	133		9	0.604	85	1.5
**X**	17		46	102		12	0.539	71	1.0

^a^ value of particle sizes for dominated fraction; ^b^ standard deviation are presented in S10, S11; ^c^ average value due to non-dominated fraction; * due to the presence of two hydrophilic units, the content of MPEGMA is given.

## Supplementary Materials

The following are available online at https://www.mdpi.com/2073-4360/12/2/330/s1, Table S1: Possibilities of encapsulation and release of selected active substances by the obtained copolymers. Table S2: DLS characteristics of empty micelles. Figure S1: ESI-MS spectra of 4nBREBr_2_. Figure S2: ^13^C NMR spectra of 4nBREBr_2_. Figure S3: Comparison of total DP of obtained P(AlHEMA–*co*–MMA) copolymers depending on the initiating group, where EiB-Br: ethyl 2-bromoisobutyrate, RET-Br: bromoester modified retinol, 4nBREBr_2_: bromoester modified 4-*n*-butylresorcinol. Figure S4: The “click” reaction efficiency depending on the type of initiating group and the percentage of AlHEMA in the copolymer. Figure S5: Plots of intensity I_336_/I_332_ ratio as a function of the logarithm of copolymers concentration in aqueous solution. Figure S6: Size distribution plots by intensity for empty polymer micelles in PBS at 25 °C. Figure S7: Size distribution plots by volume for empty polymer micelles in PBS at 25 °C. Figure S8: TEM images of sample II (a), and thin film of polymer IVc (b,c), where green rectangle in photo (b) is magnified in photo (c). Series of higher magnification of Figure 7b (d–f), where green rectangle in photo (d) is magnified in photo (e), and then green rectangle in photo (e) in magnified in photo (f). Figure S9: TEM images of microparticles formed by polymer IVc. Figure S10: Size distribution plots by intensity for VitE (a), ARB (b), or VitC (c) loaded polymer micelles in PBS at 25 °C. Figure S11: Size distribution plots by volume for VitE (a), ARB (b), or VitC (c) loaded polymer micelles in PBS at 25 °C.
