# Supplementary material for: 4-n-Butylresorcinol-Based Linear and Graft Polymethacrylates for Arbutin and Vitamins Delivery by Micellar Systems
Source: Polymers (Basel). 2020 Feb 5;12(2):330. doi: 10.3390/polym12020330 (PMC7077416; doi:10.3390/polym12020330)
Supplement: Supplementary file 1 [file polymers-12-00330-s001.pdf]

4-n-Butylresorcinol-based linear and graft polymethacrylates forming micelles for arbutin and vitamins delivery

Justyna Odrobińska[1], Łukasz Mielańczyk[2] and Dorota Neugebauer[1]*

[1] Department of Physical Chemistry and Technology of Polymers, Faculty of Chemistry, Silesian University of Technology, 44-100 Gliwice, POLAND

[2] Department of Histology and Cell Pathology, Faculty of Medical Sciences in Zabrze, Medical University of Silesia, Katowice, POLAND

* Correspondence: dorota.neugebauer@polsl.pl

Table S1. Possibilities of encapsulation and release of selected active substances by the obtained copolymers.

	VitC		VitE		ARB	
	**load**	**rel**	**load**	**rel**	**load**	**rel**
**P(HEMA-*co*-MMA)**	-	-	+	+		
**P(MMA-*co*-(HEMA-*graft*-PEG))**	-	-			+	+
**P(HEMA-*co*-MPEGMA)**	+	+	+/-	-		
**P(MMA-*co*-MPEGMA)**	+	+	+/-	-		

Table S2. DLS characteristics of empty micelles.

	D_h[a] [nm]		PDI
	by intensity	by volume	
**I**	1338	1383	1.000
**II**	73[b]	1.4	0.819
**III**	546	556[b]	0.386
**IVc**	221	40	0.253
**Vc**	227	27	0.315
**VII**	47[b]	9	0.460
**IX**	15[b]	7	0.653
**X**	23[b]	13	0.697

[a] value of particle sizes for dominated fraction; [b] average value due to non-dominated fraction;

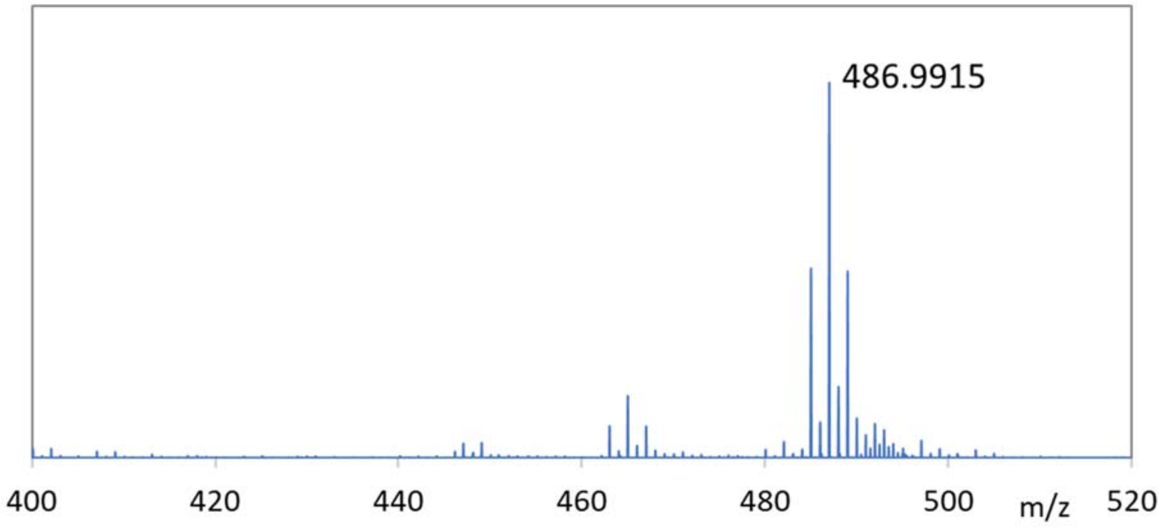

Figure S1. ESI-MS spectra of 4nBREBr2.

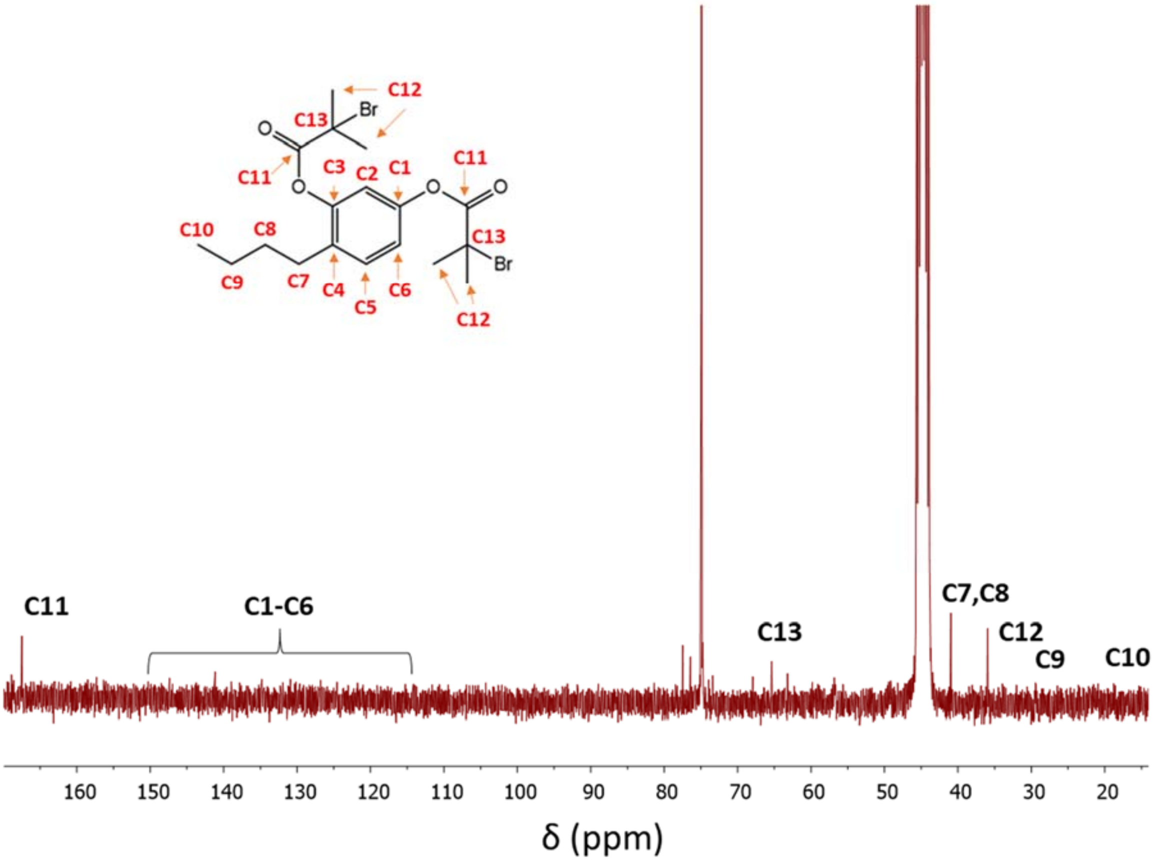

Figure S2. ^{13}C NMR spectra of 4nBREBr2.

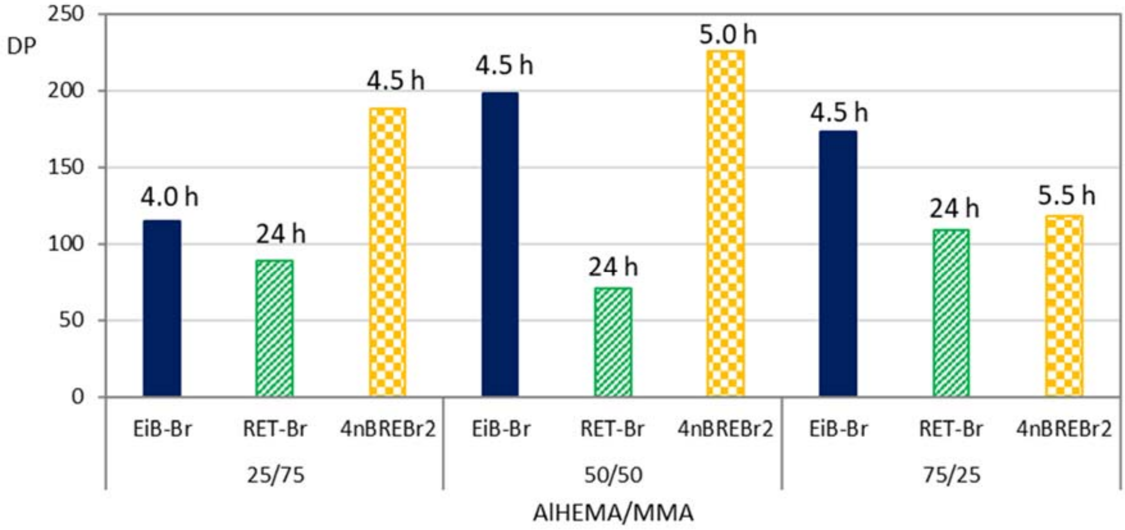

Figure S1. Comparison of total DP of obtained P(AlHEMA-*co*-MMA) copolymers depending on the initiating group, where EiB-Br: ethyl 2-bromoisobutyrate, RET-Br: bromoester modified retinol, $4nBREBr_2$: bromoester modified 4-n-butylresorcinol.

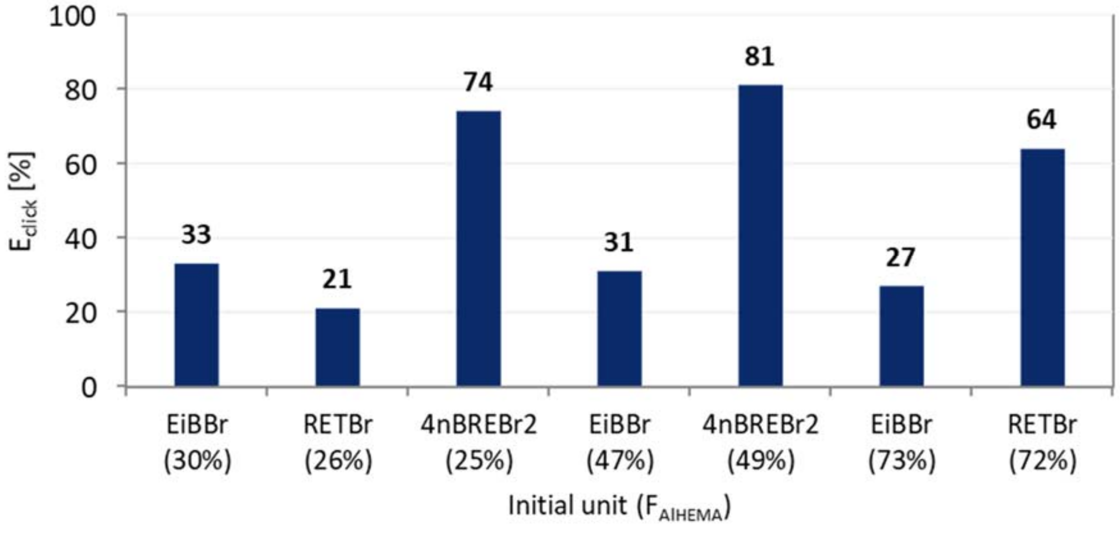

Figure S2. The "click" reaction efficiency depending on the type of initiating group and the percentage of AlHEMA in the copolymer.

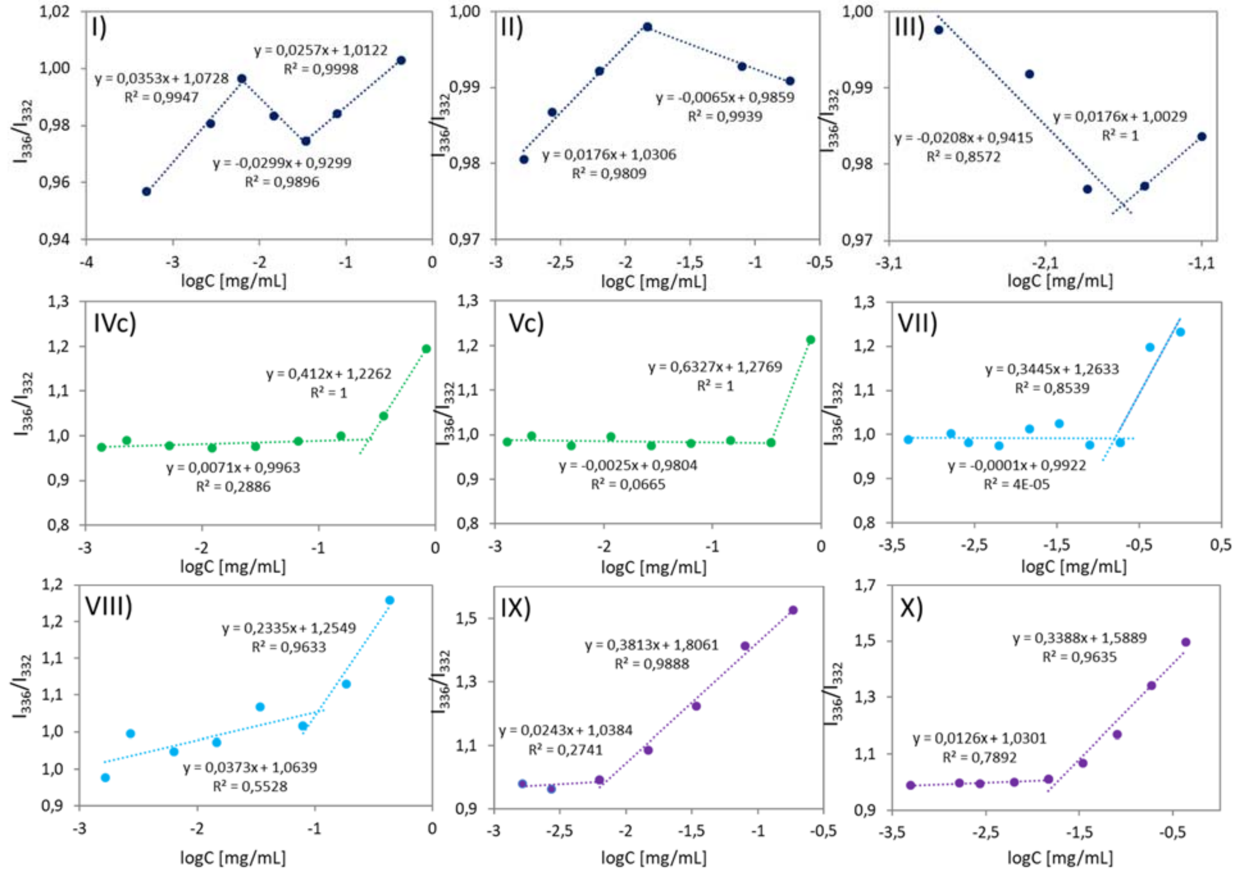

Figure S3. Plots of intensity I_{336}/I_{332} ratio as a function of the logarithm of copolymers concentration in aqueous solution.

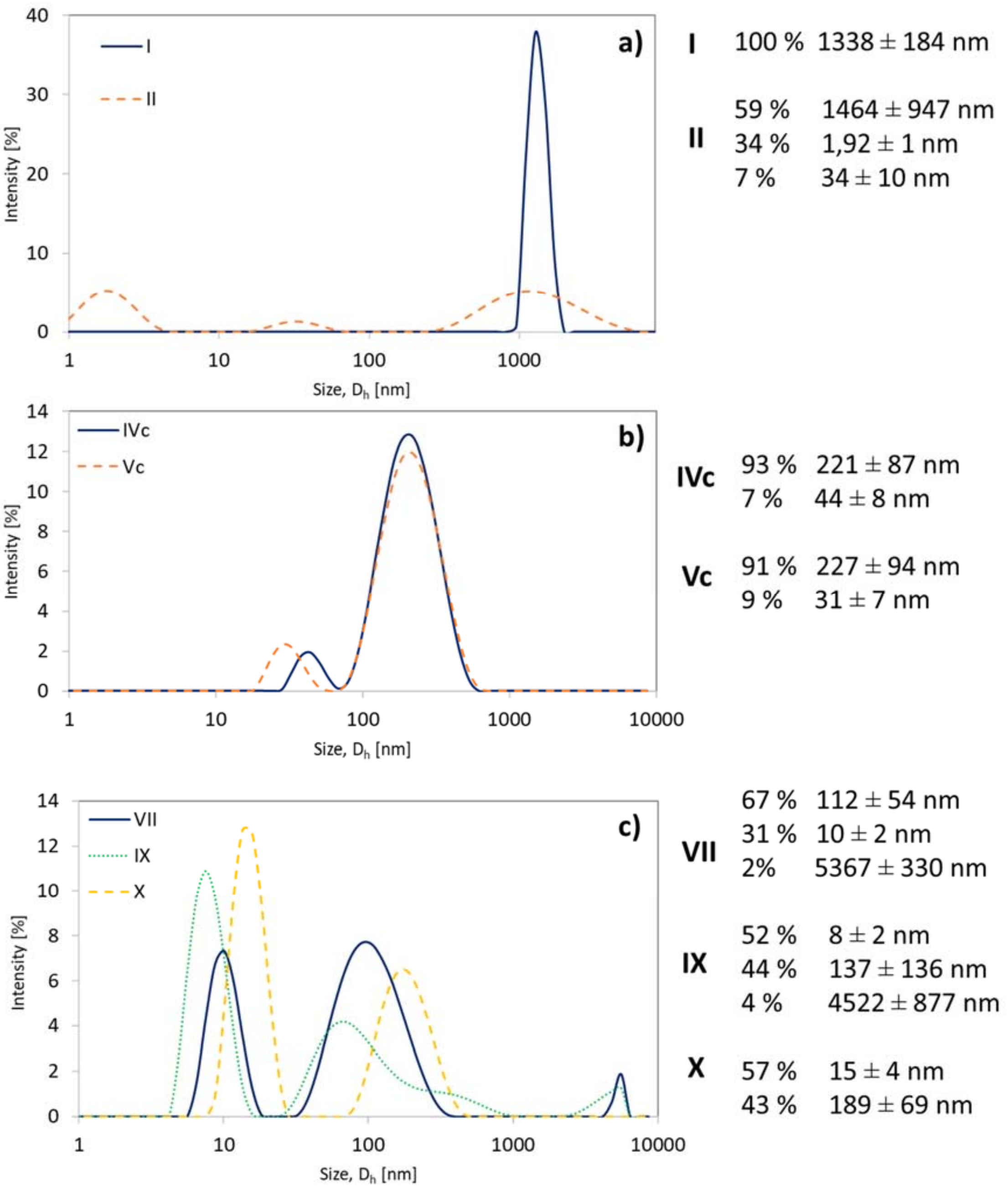

Figure S6. Size distribution plots by intensity for empty polymer micelles in PBS at 25ºC.

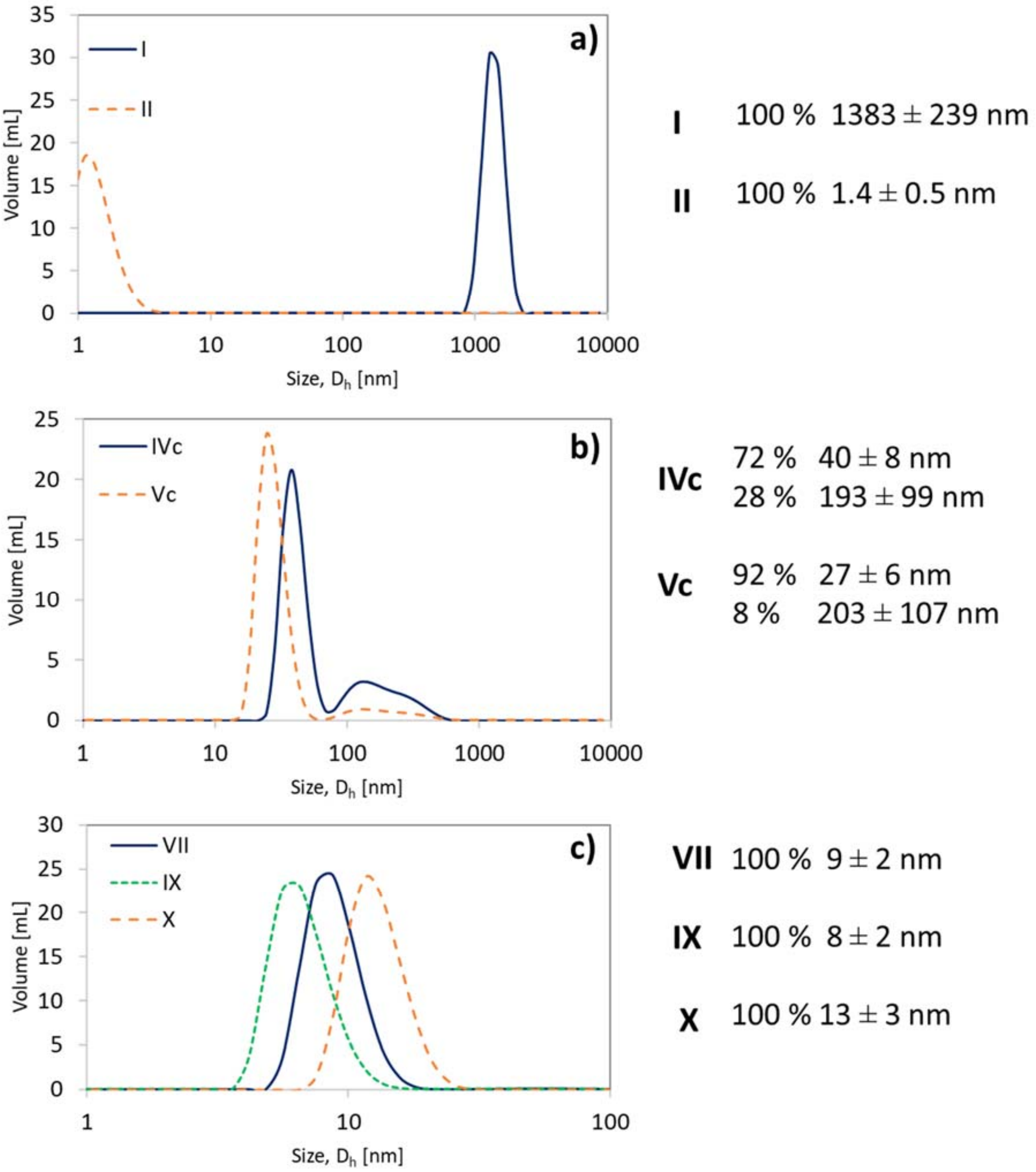

Figure S7. Size distribution plots by volume for empty polymer micelles in PBS at 25ºC.

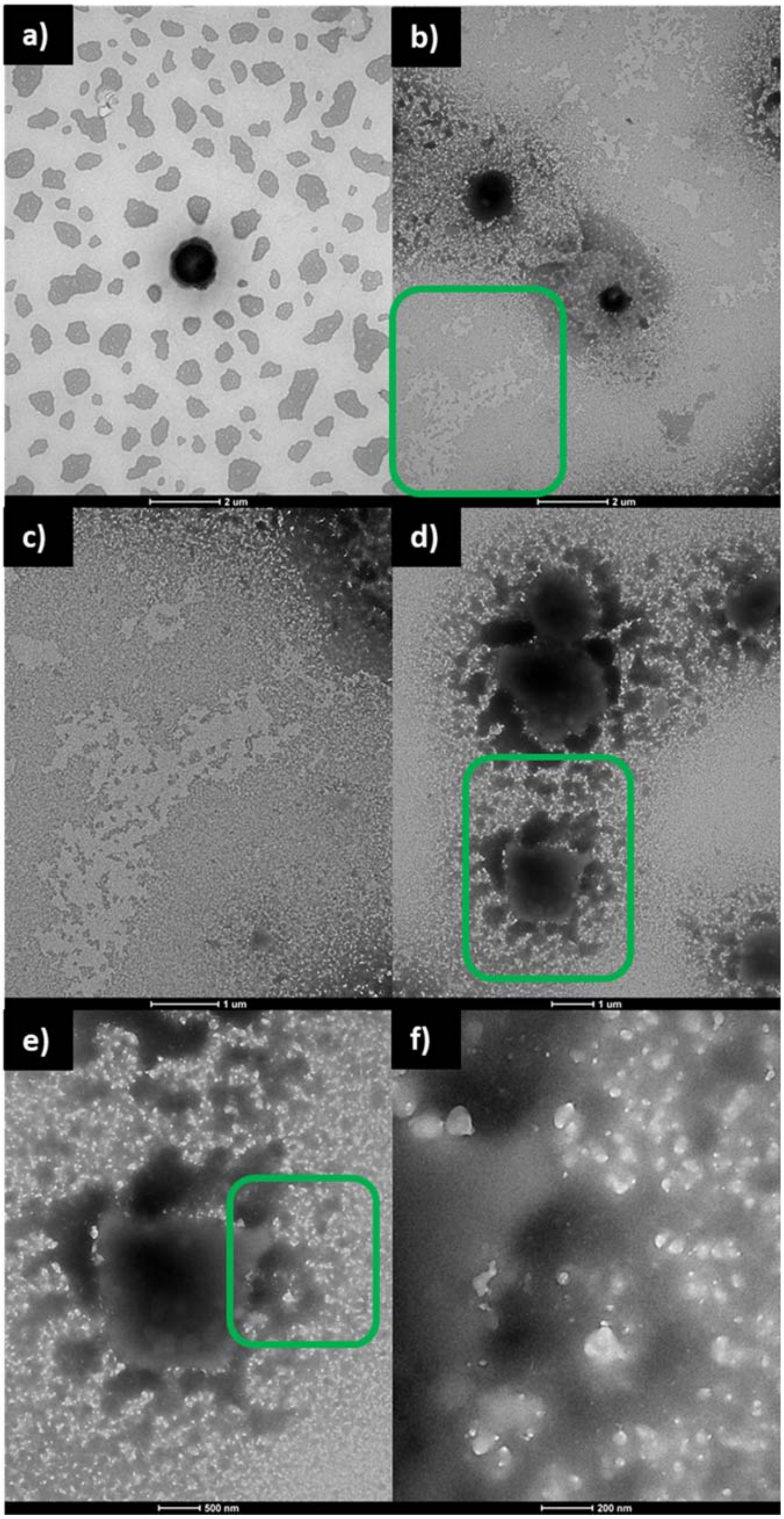

Figure S8. TEM images of sample II (a), and thin film of polymer IVc (b-c), where green rectangle in photo (b) is magnified in photo (c). Series of higher magnification of Fig. 7b (d-f), where green rectangle in photo (d) is magnified in photo (e), and then green rectangle in photo (e) in magnified in photo (f).

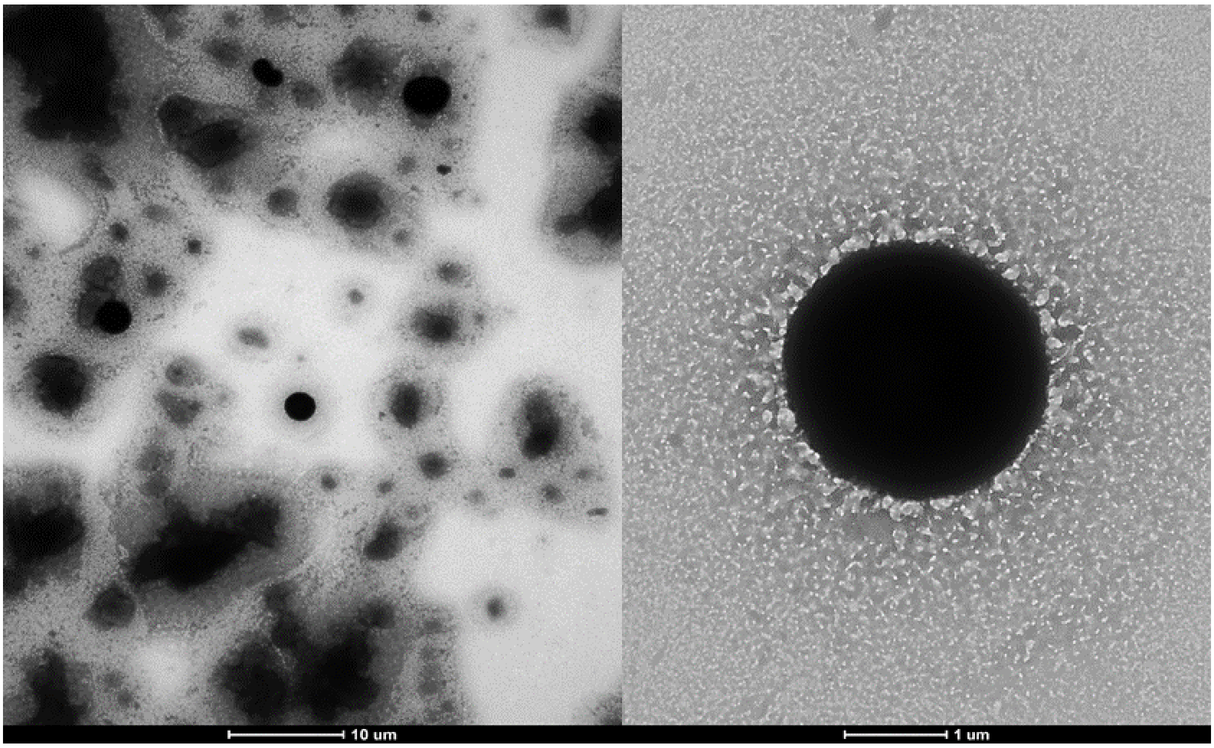

Figure S9. TEM images of microparticles formed by polymer IVc.

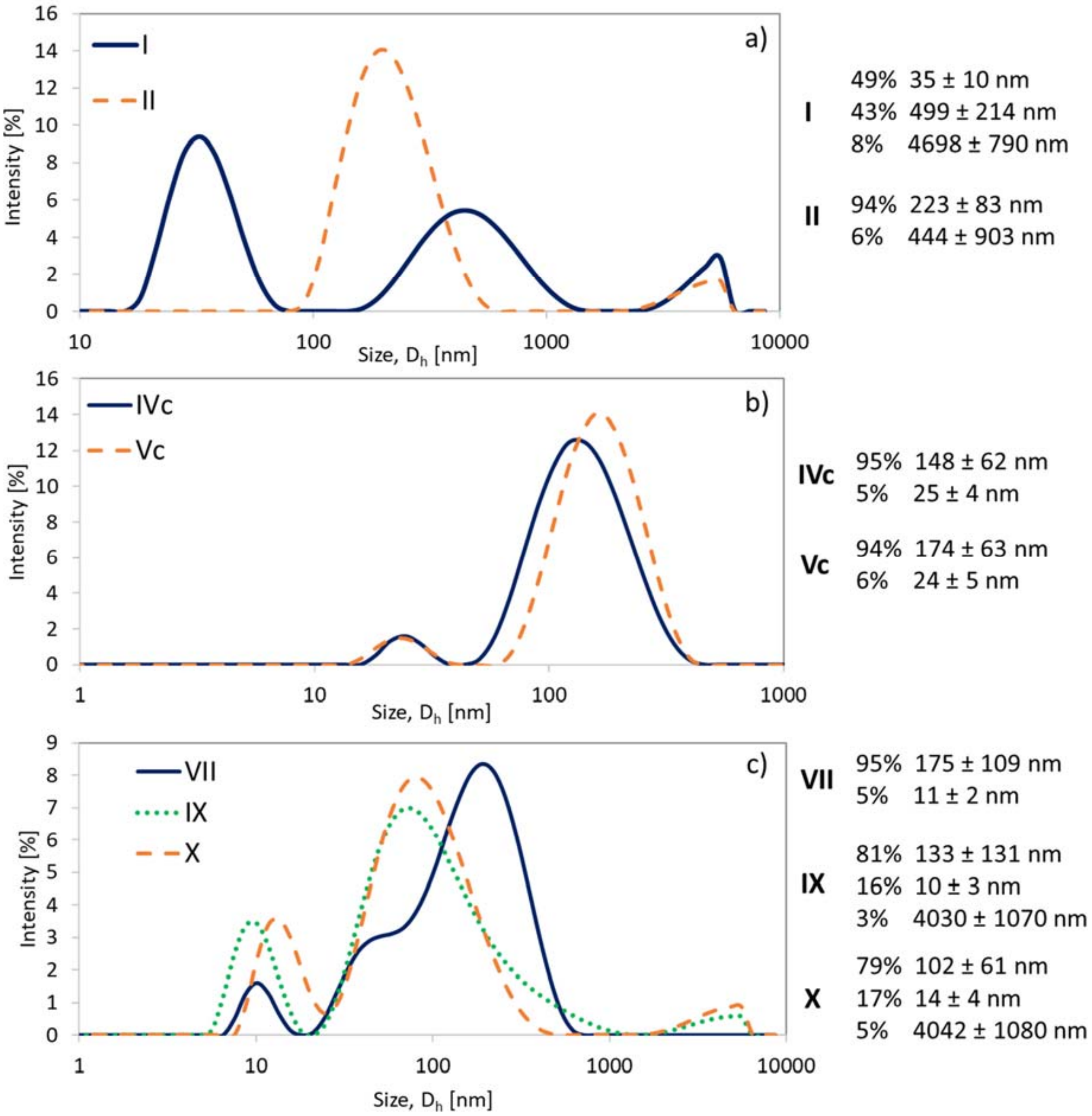

Figure S10. Size distribution plots by intensity for VitE (a), ARB (b) or VitC (c) loaded polymer micelles in PBS at 25ºC.

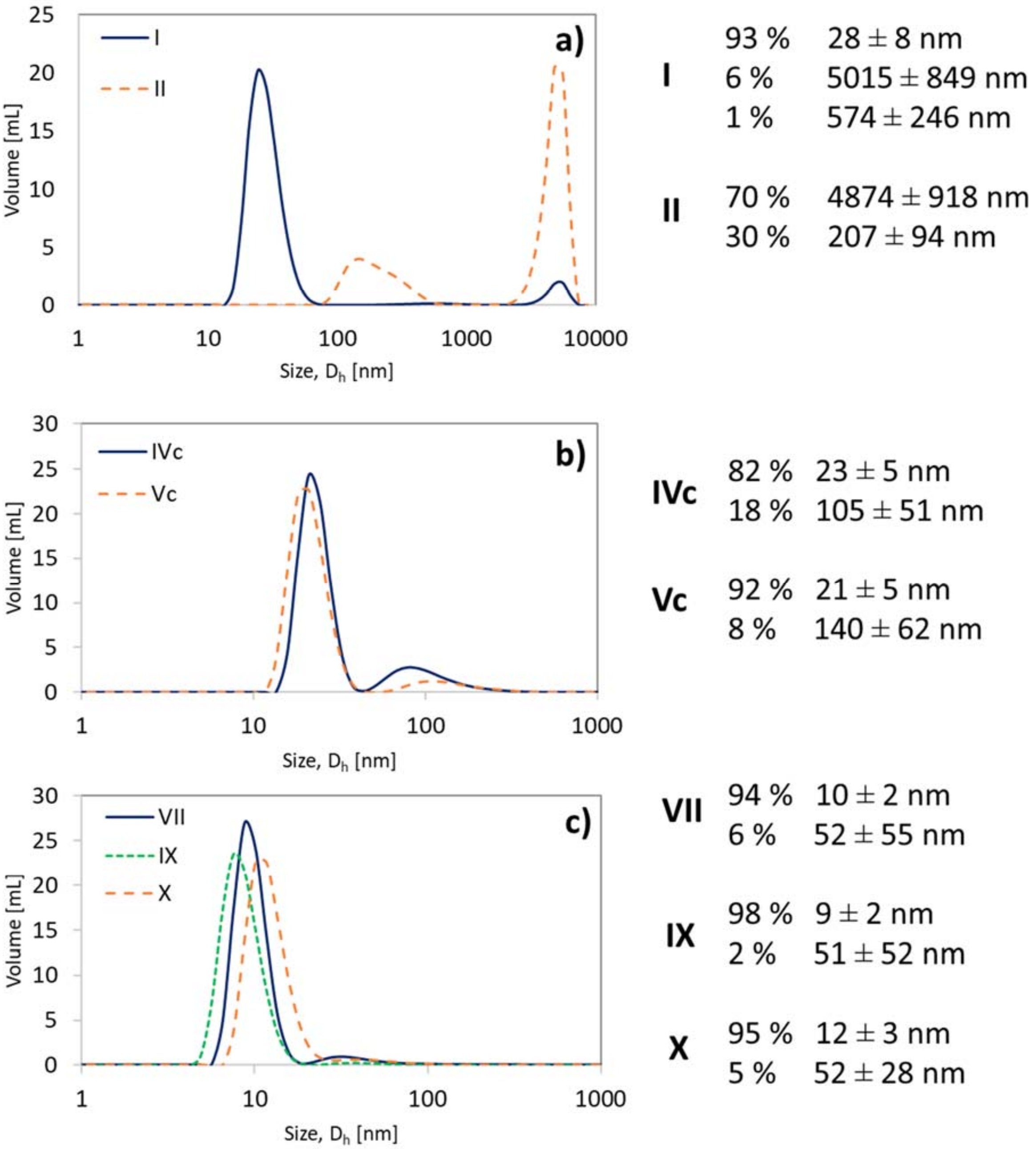

Figure S11. Size distribution plots by volume for VitE (a), ARB (b) or VitC (c) loaded polymer micelles in PBS at 25ºC.
